# Antimicrobial Activity of Actinomycetes Isolated From Hot Springs in Western Uganda

**DOI:** 10.7759/cureus.92917

**Published:** 2025-09-22

**Authors:** Joel Bazira, Karuhanga Naume Joyce, Nalumaga Pauline Petra, James Mwesigye, Kennedy Kassaza, Kawuma Simon

**Affiliations:** 1 Microbiology and Parasitology, Mbarara University of Science and Technology, Mbarara, UGA; 2 Microbiology, Mbarara University of Science and Technology, Mbarara, UGA; 3 Software and Informatics Engineering, Mbarara University of Science and Technology, Mbarara, UGA

**Keywords:** actinomycetes, antibacterial activity, antifungal activity, antimicrobial resistance, hotsprings

## Abstract

Introduction: Actinomycetes are Gram-positive filamentous bacteria belonging to the phylum Actinobacteria. This study aimed to isolate and characterize *Actinomycetes* from Ugandan hot springs and evaluate their antimicrobial activity against pathogenic microorganisms.

Materials and methods: A total of 30 *Actinomycetes* isolates were retrieved from two major hot springs in Uganda and identified using morphological and molecular techniques, including PCR amplification of the 16S rRNA gene and whole-genome sequencing. Antimicrobial activity was assessed against *Staphylococcus aureus*, *Escherichia coli*, and *Candida* species using cross-streak and agar well diffusion methods.

Results: Out of the 30 *Actinomycetes* isolates, 15 (50%) exhibited antimicrobial activity against at least one of the test pathogens; six isolates (20%) exhibited antibacterial activity against Gram-negative bacteria, four isolates (13.3%) showed activity against Gram-negative bacteria, while five isolates (16.7%) showed activity against *Candida* isolates. HB003 and HB026 were the most potent isolates against Gram-negative and Gram-positive isolates, respectively. Upon comprehensive full-genome analysis using the PATRIC software system (Virginia Bioinformatics Institute, Blacksburg, USA), the annotations of all the isolates were identified as *Actinomycete *sp.

Conclusion: This study emphasizes the potential of *Actinomycetes* from hot springs in Uganda as promising sources of bioactive compounds for developing drugs against pathogenic microorganisms, addressing the global threat of drug resistance.

## Introduction

*Actinomycetes* is a genus of Gram-positive filamentous bacteria known for their filamentous growth pattern that belongs to the phylum Actinobacteria [1]. These bacteria are widely known for having the ability to release a variety of secondary metabolites, particularly antibiotics known to combat bacterial, fungal, and parasitic infections [2,3].

Antimicrobial resistance (AMR) is one of the most critical public health challenges of the 21st century, threatening effective treatment of infectious diseases globally [4]. The emergence of drug resistance and evolving multidrug-resistant (MDR) pathogenic bacteria has rendered the currently available antibiotics increasingly less effective against available drugs [3]. According to the World Health Organization (WHO), AMR is responsible for approximately 1.27 million deaths annually, with infections caused by resistant bacteria contributing to nearly five million deaths globally each year (4). The emergence of MDR and extensively drug-resistant (XDR) pathogens, such as *Escherichia coli*, *Klebsiella pneumoniae*, and methicillin-resistant *Staphylococcus aureus *(MRSA), has significantly reduced the efficacy of existing antibiotics, leading to prolonged hospital stays, increased healthcare costs, and higher mortality rates. Addressing this crisis requires exploration of novel environments and sources of bioactive compounds, particularly from microorganisms with remarkable metabolic capabilities such as *Actinomycetes*, which are prolific producers of antibiotics and other therapeutics [5,6].

Geothermal ecosystems, defined by high temperatures, severe pH levels, and mineral-rich conditions, provide a habitat for thermophilic and extremophilic microbes. This unique environment is thought to promote microbial adaptation and synthesis of metabolites with potential antimicrobial properties [7]. *Actinomycetes* have been discovered in various hot spring environments across the world, including those in Africa [8]. In Africa, hot springs are widely distributed across several countries, including Uganda, Kenya, Tanzania, and Ethiopia, as well as in all other regions with geothermal activity [9]. Hot springs throughout the world, such as in Iceland and China, have been studied and found to be repositories of microbial diversity and biosynthetic potential [10].

Despite growing global awareness of geothermal habitats as potential sources of antimicrobial compounds, Uganda's hot springs remain largely unexplored due to challenges such as logistical constraints in accessing remote geothermal sites, inadequate funding for microbiological and genomic studies, and a lack of advanced screening technologies for bioactive compounds. Therefore, the intention of this study was to isolate *Actinomycetes* from geothermal environments to evaluate antimicrobial activity against clinically relevant pathogens and explore the genes responsible for the production of antimicrobial compounds.

## Materials and methods

Study location

This was an experimental, laboratory-based study conducted at the Department of Medical Microbiology of Mbarara University of Science and Technology (MUST), located in Southwestern Uganda, East Africa.

Laboratory methods

Sample Collection

A total of 30 *Actinomycetes *isolates, previously obtained from soil samples collected around the geothermal environments of Kitagata hot springs in Bushenyi District and Kabale hot springs in Southwestern Uganda, were stored at -70°C in the Microbiology Laboratory at MUST for six months and later retrieved following standard operating procedures. During primary isolation, the soil samples were pretreated following the method of Hayakawa et al. (1991) to suppress fast-growing bacteria and fungi [27]. Briefly, 1 g of soil was suspended in 10 mL of sterile saline, vortexed, and left to settle for 30 minutes. The suspension was then serially diluted, and 100 µL aliquots were plated onto starch casein agar. Plates were incubated at 37°C for five days, after which colonies with the characteristic chalky and filamentous morphology of *Actinomycetes* were picked, purified, and preserved at -70°C until further analysis.

Identification of *Actinomycetes*

Morphology Characterization of Actinomycetes

These isolates were thawed and sub-cultured on Starch M protein agar (HiMedia, Thane, India), a selective medium optimized for *Actinomycetes* growth and promotes colony formation [11], for seven days at 55°C. Morphological characterization of *Actinomycetes* isolates was done according to the ISP (International Streptomyces Project) recommendations [12]. Microscopic identification was done by Gram staining and observing under the 100× magnification of a light microscope.

Molecular Identification of Actinomycetes Isolates

DNA extraction: Genomic DNA was extracted using the cetyltrimethylammonium bromide (CTAB) method. DNA was purified using chloroform-isoamyl alcohol and precipitated with ethanol and stored at −20°C for further analysis.

Polymerase chain reaction (PCR) conditions: PCR amplification targeted the 16S rRNA gene using primers 63F (5′-CAG GCC TAA CAC ATG CAA GTC-3′) and 1378R (5′-GGG CGG WGT GTA CAA GGC-3′), yielding a 1300 bp product. PCR was performed in a BIORAD CFX 96 thermocycler under the following conditions: (i) initial denaturation at 95°C for five minutes, followed by cycling by 30 cycles at 95°C for one minute; (ii) annealing at 58°C for 40 seconds; and (iii) extension at 72°C for one minute and final extension at 72°C for 10 minutes. PCR products were visualized via gel electrophoresis (1% agarose gel) with a molecular ladder (1500 bp) to confirm the expected amplicon size.

Sequencing protocols: Amplicons were purified and sequenced using the Sanger method. The resulting sequences were compared against the NCBI GenBank database using BLAST to identify the *Actinomycetes* species.

Determination of antibacterial and antifungal activity of *Actinomycetes*

Primary Screening of Actinomycetes

Primary screening for the determination of antimicrobial activity was performed using the cross-streak method [13]. This technique was used to aid in identifying the *Actinomycetes* antimicrobial activity against standard strains of *Escherichia coli* ATCC 25922,* E. coli* resistant to extended-spectrum beta-lactamase (ESBL) enzyme harboring *CTX*, *TEM*, and *SHV* genes, *Staphylococcus aureus* ATCC 25904, MRSA strain, *Candida albicans* ATCC 10231, and azole-resistant *Candida famata* with the Erg 11 gene mutation. Starch M agar plates were prepared and inoculated with the pure culture of *Actinomycetes* by a single streak in the center of the Petri dish and incubated at 37°C for four days. The plates were inoculated with the test organisms by a perpendicular streak to the line of *Actinomycetes* growth. The plates were then incubated for 24 hours to determine zones of inhibition [14].

Secondary Screening of Actinomycetes

Secondary screening of *Actinomycetes* aided in analyzing the byproducts with antimicrobial products. Isolates demonstrating significant inhibition in primary screening were selected for secondary screening. Selection criteria included the presence and size of inhibition zones (≥10 mm in diameter). A pure culture of *Actinomycetes* grown on starch M agar plates for four days was inoculated into Soya Bean Casein Digest Broth, 15 mL in a 50 mL Falcon tube, using a sterile wire loop, and incubated for 24 hours. Fermentation was conducted in Soya Bean Casein Digest Broth (150 mL) at 28°C in a shaking incubator (300 rpm) for five days. Metabolites were extracted using ethyl acetate and dried to obtain crude extracts. Antimicrobial activity was evaluated using the agar well diffusion method [15].

Media Preparation and Incubation

Selective media such as Starch M protein agar and Soya Bean Casein Digest agar were prepared per the manufacturer's instructions. Starch M protein agar plates were used for initial culturing (5-10 days, 55°C), while fermentation utilized liquid broth in Erlenmeyer flasks incubated at 28°C with agitation. Indicator organisms for antimicrobial assays were cultured on Mueller-Hinton Agar for bacteria [16] and Sabouraud Dextrose Agar for the yeasts [17] at 37°C.

Quality Control

Positive controls included ciprofloxacin and fluconazole for bacterial and fungal assays, respectively. The results were interpreted according to the Clinical and Laboratory Standards Institute (CLSI) 2024 guidelines. Laboratory guidelines and protocols were used while performing all laboratory procedures.

Data management

All results obtained were double-checked for verification. Data were coded and entered into a pre-designed template in Microsoft Excel (Microsoft Corporation, Redmond, Washington) and then exported to STATA software (StataCorp. 2023. Stata Statistical Software: Release 18. College Station, TX: StataCorp LLC). The outcomes were presented in terms of percentages and discrete numbers.

Ethical consideration

The study proposal was submitted to the MUST Research Ethics Committee (REC) for approval. Permission to use the stored *Streptomyces* isolates was obtained from the principal investigator of the MUST-PES study.

## Results

Isolation and identification of the possible *Actinomycetes* from Ugandan hot springs

**Figure 1:**
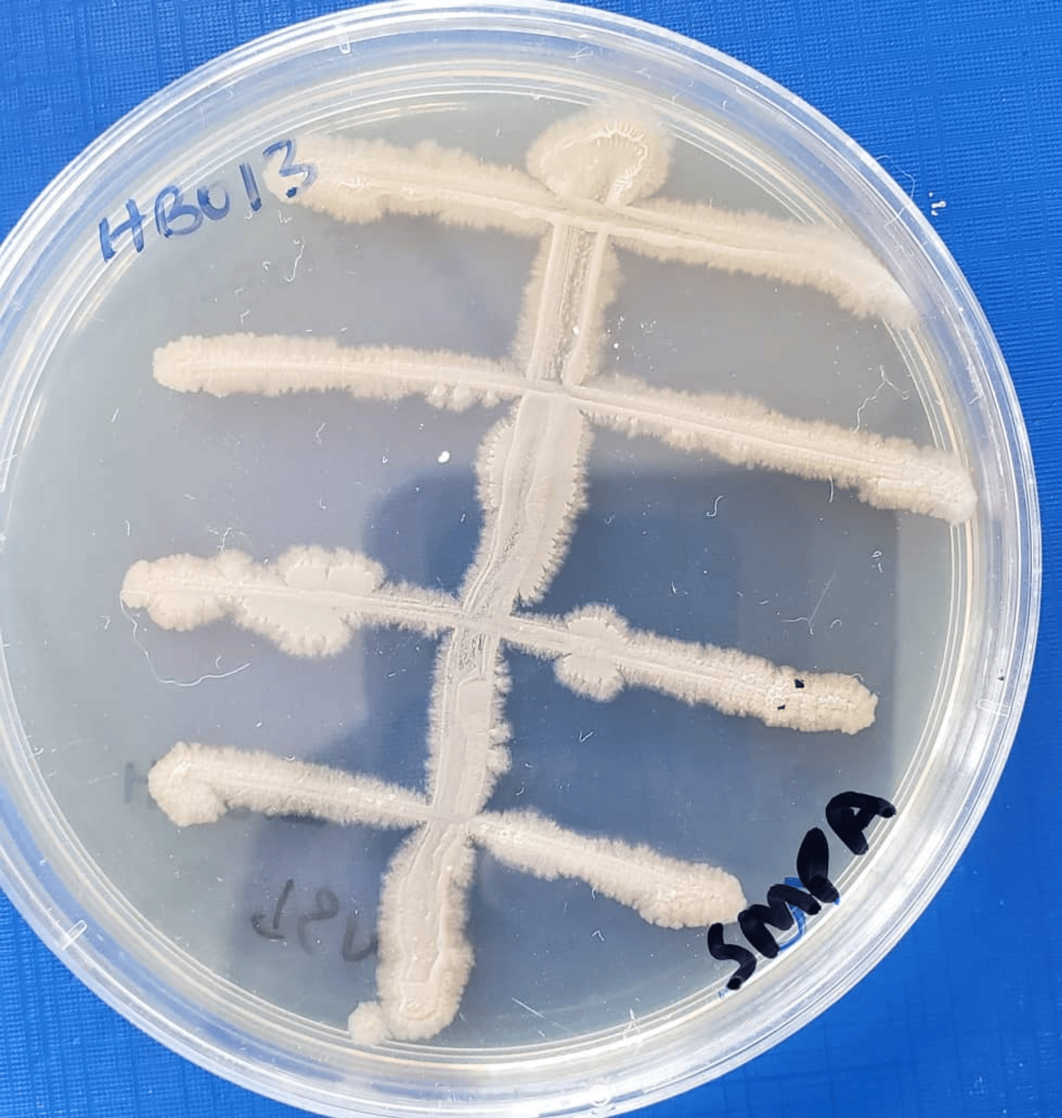
Growth of isolate HB013 from Kitagata hot spring in Bushenyi

Out of 30 isolates, 13 (43%) showed white colonies, eight (27%) yellow, seven (23%) orange, and two (7%) cream. The majority of the isolates (23, 77%) lacked mycelium production, while only seven (23%) showed mycelium production on selected culture media (Table 1, Figure 1).

**Table 1:** Morphology identification of Actinomycetes HK: *Actinomycetes* isolated from the Kabale hot spring HB: *Actinomycetes* isolated from Kitagata hot spring in Bushenyi

Sample	Source of Isolate	Color of Colonies	Mycelium Production
HKOO9-2	Kabale Hot Spring	White	Yes
HB022	Kitagata Hot Spring – Bushenyi	White	Yes
HB010	Kitagata Hot Spring – Bushenyi	Yellow	Yes
HB030	Kitagata Hot Spring – Bushenyi	White	No
HB013	Kitagata Hot Spring – Bushenyi	White	Yes
HB012-2	Kitagata Hot Spring – Bushenyi	Yellow	No
HB003	Kitagata Hot Spring – Bushenyi	White	No
HB004	Kitagata Hot Spring – Bushenyi	Orange	No
HB028-2	Kitagata Hot Spring – Bushenyi	Yellow	No
HB028-1	Kitagata Hot Spring – Bushenyi	Yellow	No
HK012	Kabale Hot Spring	White	No
HB032	Kitagata Hot Spring – Bushenyi	Orange	No
HB005	Kitagata Hot Spring – Bushenyi	Orange	No
HB021	Kitagata Hot Spring – Bushenyi	White	Yes
HK009-1	Kabale Hot Spring	White	No
HB014	Kitagata Hot Spring – Bushenyi	White	Yes
HKO10	Kabale Hot Spring	Cream	No
HKOO8	Kabale Hot Spring	White	Yes
HB016	Kitagata Hot Spring – Bushenyi	Orange	No
HB009	Kitagata Hot Spring – Bushenyi	Yellow	No
HB033	Kitagata Hot Spring – Bushenyi	Orange	No
HB029-1	Kitagata Hot Spring – Bushenyi	Cream	No
HB027	Kitagata Hot Spring – Bushenyi	Orange	No
HB025	Kitagata Hot Spring – Bushenyi	White	No
HB015	Kitagata Hot Spring – Bushenyi	Orange	No
HB031	Kitagata Hot Spring – Bushenyi	Yellow	No
HB024	Kitagata Hot Spring – Bushenyi	White	No
HB007-1	Kitagata Hot Spring – Bushenyi	Yellow	No
HB026	Kitagata Hot Spring – Bushenyi	White	No
Total	-	30	30

Molecular identification of *Actinomycetes* isolates

Genotypic Identification of the Isolates

**Figure 2:**
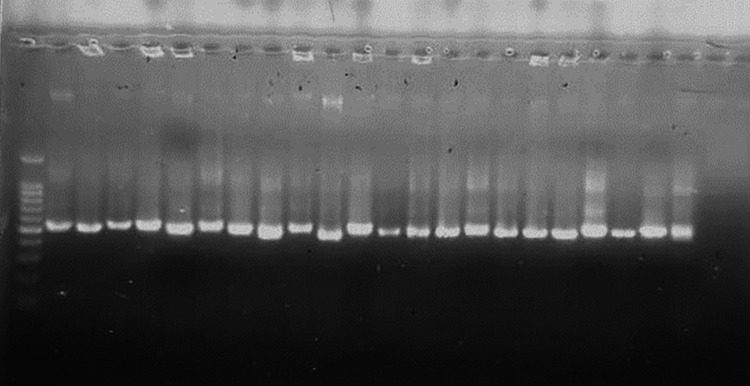
Electrophoresis gel image of Actinomycetes

PCR amplification of the 16S rRNA gene produced clear ~1300 bp bands in all isolates, as shown in Figure 2.

Whole-Genome Sequencing

**Figure 3:**
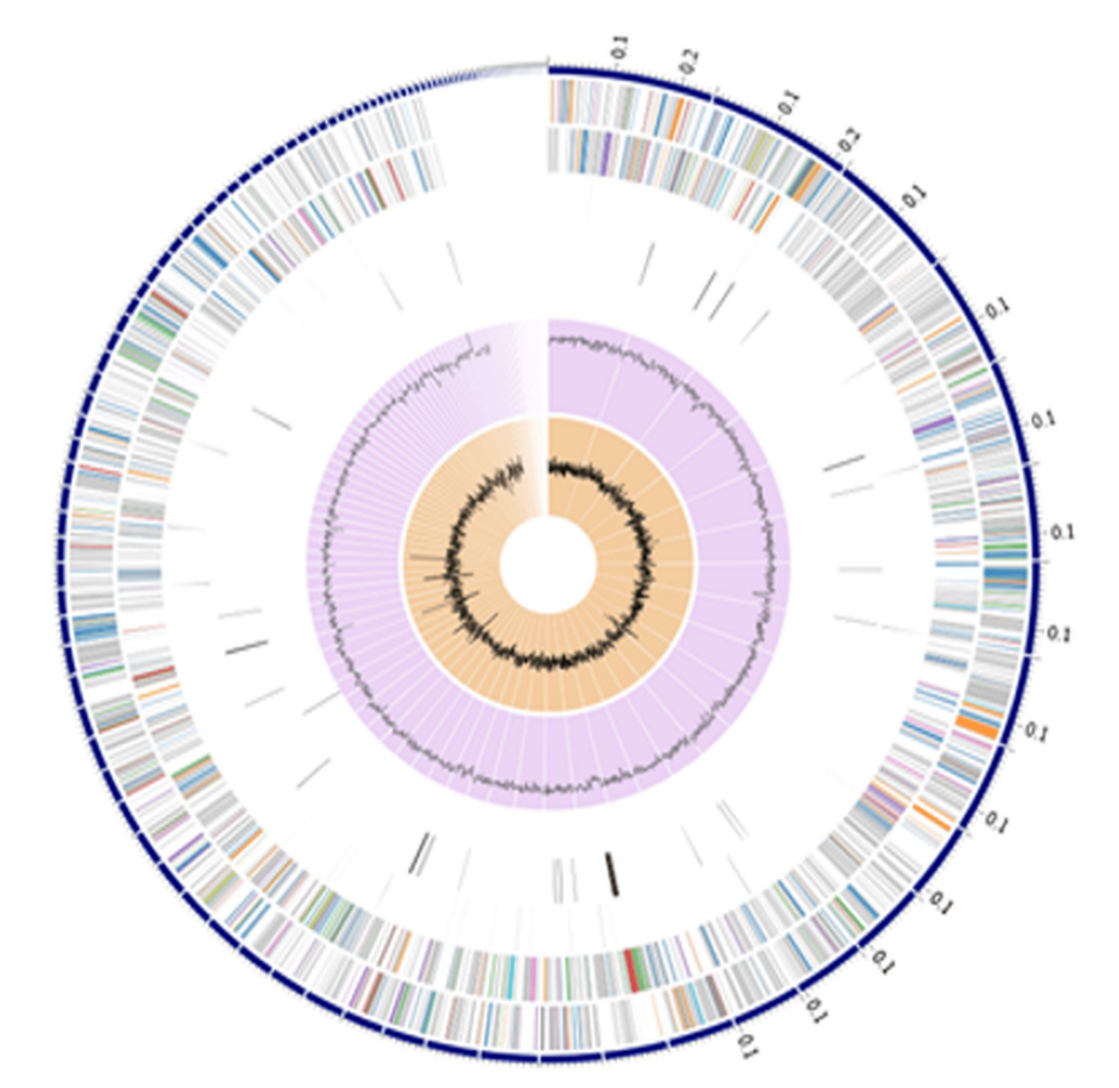
Graphical display of the distribution of genome annotations of sequenced strains From outer to inner rings, contigs, coding sequences (CDS) on the forward strand, CDS on the reverse strand, RNA genes, CDS with antimicrobial resistance genes, CDS with virulence factors, GC content, and GC skew. The colors on CDS on the forward and reverse strands indicate the subsystem where the functionality proteins belong. Image created by the authors.

**Figure 4:**
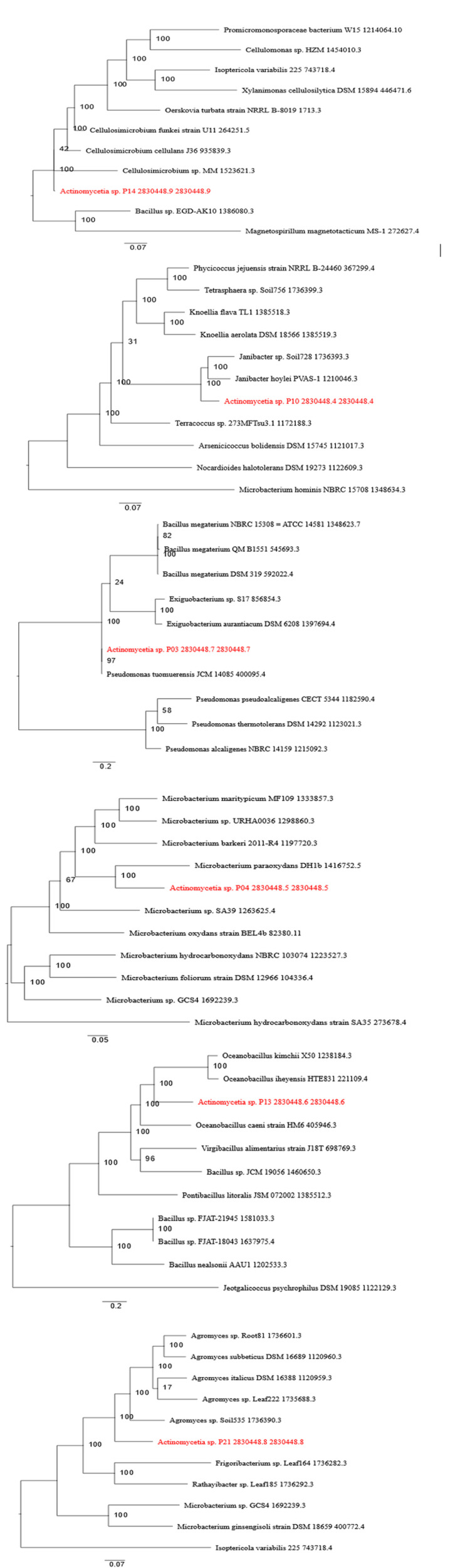
Evolutionary distance tree showing phylogenetic placement of sequenced isolates All the isolates exhibited >99% sequence homology with *Acticetianomycetia *sp. Image created by the authors.

Whole-genome sequencing of selected isolates revealed high GC content (53.94%-74.55%) and identified various AMR genes. Isolate P03 harbored the highest number of AMR genes (145), including genes encoding efflux pumps and antibiotic-modifying enzymes. Phylogenetic analysis confirmed that all isolates were distinct members of the Actinomycetia class (Tables 2, 3) (Figures 3, 4). This enabled the identification of biosynthetic gene clusters (BGCs) associated with antimicrobial compound production, confirming the metabolic potential of the isolated *Actinomycetes*.

**Table 2:** Genome assembly features of sequenced isolates Isolates labeled with P represent *Actinomycete* isolates.

Genome Features	Isolate Identification			
	P03	P04	P13	P21
Contigs	677	641	87	36
GC content (%)	53.94	57.37	37.6	72.28
Contig L50	40	81	9	8
Contig N50	114,697	28,400	173,000	147,808
Genome length	15,704,711 bp	7,722,833 bp	4,336,595 bp	3,218,242 bp
Genome annotation	Actinomycetia sp.	Actinomycetia sp.	Actinomycetia sp.	Actinomycetia sp.
					Unique genome identity	2830448.7	2830448.5	2830448.6	2830448.8

**Table 3:** Antimicrobial resistance genes and their mode of action in selected Actinomycetes P represents selected *Actinomycetes* isolates.

AMR Mechanism	Genes in Selected Isolates					
	P10	P14	P03	P04	P13	P21
Antibiotic activation enzyme	-	-	KatG	KatG	-	-
Antibiotic inactivation enzyme	AAC(3)-I	-	CatA family, FosB, Vgb(A)	APH(6)-Ia/APH(6)-Ib	FosB	-
Antibiotic target in susceptible species	Alr, Ddl, dxr, EF-G, EF-Tu, folA, Dfr, folP, gyrA, gyrB, inhA, fabI, Iso-tRNA, kasA, rho, rpoB, rpoC, S10p, S12p	Alr, Ddl, dxr, EF-G, EF-Tu, folA, Dfr, folP, gyrA, gyrB, inhA, fabI, Iso-tRNA, kasA, MurA, rho, rpoB, rpoC, S10p, S12p	Alr, Ddl, dxr, EF-G, EF-Tu, folA, Dfr, folP, gyrA, gyrB, inhA, fabI, Iso-tRNA, kasA, MurA, rho, rpoB, rpoC, S10p, S12p	Alr, Ddl, dxr, EF-G, EF-Tu, folA, Dfr, folP, gyrA, gyrB, inhA, fabI, Iso-tRNA, kasA, MurA, rho, rpoB, rpoC, S10p, S12p	Alr, Ddl, EF-G, EF-Tu, folA, Dfr, folP, gyrA, gyrB, inhA, fabI, Iso-tRNA, kasA, MurA, rho, rpoB, rpoC, S10p, S12p	Alr, Ddl, dxr, EF-G, EF-Tu, folA, Dfr, folP, gyrA, gyrB, Iso-tRNA, kasA, MurA, rho, rpoB, rpoC, S10p, S12p
Antibiotic target modifying enzyme	-	-	-	Erm(X)	RlmA(II)	-
Antibiotic target protection protein	-	-	BcrC	-	-	-
Efflux pump conferring antibiotic resistance	-	-	BceA, BceB, MdtABC-OMF, MdtABC-TolC, MexJK-OprM/OpmH, MexVW-OprM, TolC/OpmH	BceA, BceB, Cmx family, Tet(42)	BceA, BceB, YkkCD	-
Antibiotic target replacement protein	FabG, HtdX	FabG, HtdX	FabG, fabL, HtdX	fabL, FabL-like	-	FabL-like
Gene conferring resistance via absence	gidB	gidB	gidB	gidB	gidB	gidB
Protein altering cell wall charge conferring antibiotic resistance	GdpD, PgsA	GdpD, PgsA	GdpD, PgsA	GdpD, PgsA	GdpD, PgsA	GdpD, PgsA
Protein modulating permeability to antibiotic	-	-	OprD family, OprF	-	-	-
Regulator modulating expression of antibiotic resistance genes	MtrA, MtrB	MtrA, MtrB	BceR, BceS, LiaF, LiaR, LiaS, MtrA, MtrB, OxyR	BceR, BceS, LiaF, LiaR, LiaS, MtrA, MtrB, VanO-type	BceR, BceS, LiaF, LiaR, LiaS	LpqB, MtrA, MtrB

 Antimicrobial activity of the *Actinomycetes* isolated from Ugandan hot springs

Primary Screening

Primary screening using the cross-streak method revealed that 15/30 (50%) of the isolates showed antimicrobial activity. Four isolates demonstrated broad-spectrum activity against both Gram-positive and Gram-negative bacteria. Six isolates were active only against Gram-positive bacteria, while five isolates were active only against Gram-negative bacteria. Notably, isolate HB003 showed the highest activity against Gram-negative bacteria (zone of clearance: 18.0 mm), while HB026 exhibited the highest activity against Gram-positive bacteria (zone of clearance: 18.0 mm), suggesting these isolates as potential candidates for further microbiology studies (Table 4).

**Table 4:** Results of primary screening against the test organisms + Activity evidenced by the zone of clearance observed - No activity observed by no zone of clearance observed HB: *Actinomycetes* isolated from Kitagata hot spring in Bushenyi HK: *Actinomycetes* isolated from Kabale hot spring

Isolate Identification Number	*Escherichia coli (*ATCC 25922)	Extended-Spectrum Beta Lactamase-Producing *E. coli*	*Staphylococcus aureus* (ATCC 25904)	Methicillin-Resistant *Staphylococcus aureus*	*Candida albicans *(ATCC 10231)	Azole-Resistant *Candida famata*
HK012	+	+	+	+	+	+
HK009-2	-	-	+	+	+	+
HBO28-2	+	+	+	+	+	+
HB030	+	+	+	+	+	+
HB013	-	-	+	+	+	+
HB021-1	-	-	-	-	+	+
HKOO9-1	+	+	+	+	+	+
HB022	-	-	-	-	+	+
HB028-1	+	+	+	+	+	+
HK010	+	+	+	+	+	+
HB003	-	-	-	-	-	-
HB004	+	+	+	+	+	+
HB005	+	+	-	-	+	+
HB010	-	-	+	+	+	+
HB032	+	+	+	+	+	+
HB012-2	+	+	+	+	+	+
HK008	+	+	+	+	+	+
HBO21	-	-	-	-	+	+
HB014	-	-	+	+	+	+
HB026	-	-	+	+	+	+

Secondary Screening

Antimicrobial activity was confirmed for all 15 isolates, with significant inhibition zones observed (p < 0.05) (Table 5). Activity against *Candida* species was observed in six isolates, with the largest zone of clearance (14.0 mm) recorded for isolate HB026.

**Table 5:** Zones of clearance by the Actinomycetes secondary metabolites HB: *Actinomycetes* isolated from Kitagata hot spring in Bushenyi HK: *Actinomycetes* isolated from Kabale hot spring ATCC: American Type Culture Collection

Identification Number	Isolate	Zone of Clearance (mm)					
								*Escherichia coli* (ATCC 25922)	Extended-spectrum beta-lactamase-producing *E. coli*	*Staphylococcus aureus* (ATCC 25904)	Methicillin-resistant *Staphylococcus aureus*	*Candida albicans* (ATCC 10231)	Azole-resistant *Candida famata*
		1	HK012	0	0	12	12	0	0
2	HK009-2	12	10	0	0	12	12
3	HBO28-2	15	15	12	12	12	12
4	HB030	15	15	0	0	10	10
5	HB021-1	0	0	19	19	0	0
6	HB022	17	16	15	14	0	0
7	HK010	10	10	0	0	0	0
8	HB003	18	16	15	15	0	0
9	HB004	0	0	12	12	0	0
10	HB005	10	10	14	14	12	12
11	HB032	0	0	18	14	0	0
12	HB012-2	0	0	16	12	0	0
13	HK008	12	10	0	0	0	0
14	HB014	10	10	0	0	10	10
15	HB026	0	0	18	16	14	14
Ciprofloxacin	-	30 mm	8 mm	30 mm	8 mm	-	-
Fluconazole	-	-	-	-	-	26 mm	8 mm

## Discussion

This study demonstrates the potential of *Actinomycetes* isolated from Ugandan hot springs as a source of novel antimicrobial compounds. Morphological, molecular, and genomic analyses confirmed that the isolates belong to the genus *Actinomycetes*, with some isolates exhibiting significant antibacterial and antifungal activities against clinically relevant pathogens. In reference to the standard positive controls, ciprofloxacin for antibacterial assays and fluconazole for antifungal assays, 15 out of 30 (50%) of the Actinomycetes isolates exhibited antimicrobial activity, suggesting a strong antimicrobial potential. This broad-spectrum activity highlights their value as promising compounds for further purification and compound identification. These findings contribute to the evidence supporting geothermal environments as reservoirs of bioactive microorganisms, aligning with similar findings in different geothermal sites worldwide [5,18-24].

The results revealed a diverse spectrum of microbial activity where 20% of isolates inhibited Gram-negative bacteria, another 13.3% inhibited Gram-positive bacteria, and 16.7% showed antifungal activity against *Candida* species. Particularly, isolates HB003 and HB026 exhibited strong antibacterial activity, with inhibition zones of 18.0 mm in diameter, comparable to and even exceeding those of resistant strains to standard antibiotics, such as ciprofloxacin. HB026 also demonstrated antifungal activity (14.0 mm) against *Candida* species, suggesting that some hot spring *Actinomycetes *may produce broad-spectrum antimicrobial compounds.

The GC content and abundance of AMR genes observed in our isolates are comparable to those reported in *Actinomycetes* from geothermal sites globally [25,26]. This genomic analysis of six selected isolates further strengthened our observations. High GC content (ranging from 53.94% to 74.55%) and the detection of BGCs and AMR genes confirm their metabolic versatility and potential to produce novel antibiotics. The presence of genes for efflux pumps, antibiotic-modifying enzymes, and target alteration suggests intrinsic defense mechanisms, possibly co-evolved in response to microbial competition in the geothermal environment, highlighting the complex genomic adaptation to extreme niches.

Hot springs are extreme environments with unique selective pressures that may drive the evolution of novel bioactive compounds not found in mesophilic microbes. Thus, isolates from such settings may be sources of new chemical compounds with activity against resistant microbes, including MRSA, ESBL-producing *Enterobacteriaceae*, and fluconazole-resistant *Candida *species, as used in this study.

However, unlike some studies employing species-level identification, our analysis focused on genus-level characterization, which limits direct strain comparisons.

Future studies should incorporate advanced molecular techniques such as metagenomics sequencing for comprehensive microbial profiling. Scaling up fermentation processes and isolating specific antimicrobial compounds through chromatographic techniques and mass spectrometry are critical next steps for harnessing the therapeutic potential of these isolates. Additionally, testing the efficacy of these compounds against a broader range of multidrug-resistant pathogens could validate their clinical relevance.

## Conclusions

The geothermal hot springs of Uganda harbor thermophilic *Actinomycetes* with significant antimicrobial potential and novel drug sources to combat pathogenic microorganisms, addressing the global challenge of antimicrobial resistance. This observation indicates the untapped microbial resources with immense potential for drug discovery. Furthermore, the unique adaptations of these microorganisms to extreme conditions provide insights into novel biochemical pathways and secondary metabolites with antimicrobial properties.
